# The Role of Biofilms in the Development and Dissemination of Microbial Resistance within the Food Industry

**DOI:** 10.3390/foods9060816

**Published:** 2020-06-21

**Authors:** Efstathios Giaouris, Manuel Simões, Florence Dubois-Brissonnet

**Affiliations:** 1 Laboratory of Biology, Microbiology and Biotechnology of Foods (LBMBF), Department of Food Science and Nutrition, School of the Environment, University of the Aegean, Ierou Lochou 10 & Makrygianni, 81400 Myrina, Lemnos, Greece; 2 Laboratory for Process Engineering, Environment, Biotechnology and Energy (LEPABE), Department of Chemical Engineering, Faculty of Engineering, University of Porto, Rua Roberto Frias, s/n, 4200-465 Porto, Portugal; mvs@fe.up.pt; 3 Micalis Institute, INRAE, AgroParisTech, Université Paris-Saclay, 78350 Jouy-en-Josas, France; florence.dubois-brissonnet@agroparistech.fr

**Keywords:** biofilms, foodborne pathogens, dairy bacilli, stress adaptation, resistance, disinfection, biocontrol, enzymes, food safety

## Abstract

Biofilms are multicellular sessile microbial communities embedded in hydrated extracellular polymeric matrices. Their formation is common in microbial life in most environments, while those formed on food-processing surfaces are of considerable interest in the context of food hygiene. Biofilm cells express properties that are distinct from planktonic ones, in particular, notorious resistance to antimicrobial agents. Thus, a special feature of biofilms is that, once they have been developed, they are hard to eradicate, even when careful sanitization procedures are regularly applied. A great deal of ongoing research has investigated how and why surface-attached microbial communities develop such resistance, and several mechanisms are to be acknowledged (e.g., heterogeneous metabolic activity, cell adaptive responses, diffusion limitations, genetic and functional diversification, and microbial interactions). The articles contained in this Special Issue deal with biofilms of some important food-related bacteria (including common pathogens such as *Salmonella enterica*, *Listeria monocytogenes*, and *Staphylococcus aureus*, as well as spoilage-causing spore-forming bacilli), providing novel insights on their resistance mechanisms and implications, together with novel methods (e.g., use of protective biofilms formed by beneficial bacteria, enzymes) that could be used to overcome such resistance and thus improve the safety of our food supply and protect public health.

The formation of biofilms spontaneously happens in both natural and industrial environments, wherever there are microorganisms, surfaces, nutrients, and water. In previous years, many studies have been occupied with detrimental biofilms, such as those formed by/containing pathogenic microorganisms, providing enough useful data on the complex mechanisms that may account for their increased recalcitrance towards antimicrobials, host immune system, and many other physicochemical stresses. Thus, diffusion limitations to the free access of some antimicrobials inside the robust biofilm matrix, variability in the physicochemical microenvironments within the biofilm (e.g., pH, oxygen levels, nutrients), cellular adaptations resulting from altered gene expression and/or horizontal gene transfer, microbial interactions, and the differentiation of biofilm-enclosed microbial cells into particularly durable variants, such as viable but not culturable (VBNC) ones, and persisters, may all account, at different levels and depending on the specific microorganisms and the surroundings, to the robustness of biofilms [1]. Their establishment as the default mode of microbial growth is hence almost everywhere. Biofilms formed by pathogenic bacteria are of special interest in the context of food hygiene since they may significantly compromise food safety [2]. Those containing spoilage microflora can downgrade food quality, limiting shelf life of the products, and induce several other important issues (e.g., clogging of membranes, increases in energy costs, biofouling, and corrosion problems). In the articles of this Special Issue, interesting data are presented regarding such biofilm communities towards the better understanding of the factors that can influence their sessile development (e.g., microbial interactions, sporulation, food residues, temperature), the mechanisms lying behind their antimicrobial resistance, together with some novel alternative methods that could be exploited to address this important problem (e.g., use of lactic acid bacteria and/or their metabolites, enzymes, bacteriophages, quorum sensing inhibitors), with lower possibilities for resistance occurrence.

*Bacillus* species are frequently encountered in the dairy processing environment and can form biofilms on surfaces containing their spores, and in this way, resist cleaning-in-place (CIP) regimes commonly applied in the dairy industry. Those consist of regular cleaning of equipment with alkaline and acidic solutions under turbulent flow conditions at high temperatures. Ostrov et al. [3] investigated the resistance of biofilm-derived spores of four dairy-associated *Bacillus* isolates (including one *B. licheniformis*, one *B. subtilis*, and two *B. paralicheniformis* strains) to CIP procedures and compared to those of a non-dairy *B. subtilis* isolate, using in parallel two different model systems simulating the typical conditions for milking systems. As cleaning solutions, they used caustic soda (0.5% w/v NaOH), sodium hypochlorite (0.018% v/v NaOCl), and six different commercial alkaline detergents commonly used in dairy farms and at concentrations recommended by the manufacturers. They observed that the dairy-associated isolates displayed increased resistance to mechanical (i.e., water circulation), chemo-biological (i.e., cleaning), and bactericidal (i.e., disinfection) effects of the tested CIP procedures compared to the non-dairy *Bacillus*. This was attributed to their robust biofilm formation and to differences in the structure and composition of their biofilm matrix resulting in its mucoid appearance. This finding was further reinforced by the enhanced resistance of two other poly-*γ*-glutamic acid (PGA)-overproducing *B. subtilis* strains to the tested CIP procedures, compared to the wild type strain. These mutant strains could indeed produce high amounts of proteinaceous extracellular matrix, which was similar in appearance to that produced by the tested dairy *Bacillus* isolates. The authors highlighted the importance of using strong biofilm-formers, such as biofilm-derived spores of dairy-associated *Bacillus*, upon evaluating the performance of commercial cleaning agents for use in industrial conditions. Undoubtedly, their results seem important towards the refinement of the industrial CIP processes to increase their efficiency in eliminating well established biofilms. 

Bovine mastitis is among the most common diseases that the dairy industry should deal with, resulting in considerable economic losses due to milk wastage and treatment costs. This is frequently caused by pathogenic staphylococci capable of forming biofilms inside the udder and making this ineffective the subsequent antibiotic therapy. Wallis et al. [4] evaluated the in vitro efficiency of an alternative therapeutic approach based on the formation of beneficial (probiotic) biofilms by lactic acid bacteria (LAB). For this, they employed five LAB strains (including three *Lactobacillus plantarum*, one *L. brevis,* and one *L. rhamnosus*) and tested them for their ability to eradicate and replace harmful *Staphylococcus* biofilms, formed by three different species all known to be implicated in bovine mastitis (i.e., *Staphylococcus aureus*, *S. xylosus*, and *S. epidermidis*). To do this, they left staphylococci to form biofilms on the wells of polypropylene 96-well plates at 37 °C for 168 h before the addition of each LAB culture and further incubation at 37 °C for 168 h. They removed biofilm cells from surfaces at three different time intervals and enumerated them. They found that all the tested LAB strains were able to remove the pathogenic biofilms, while two of them (*L. rhamnosus* ATCC 7469 and *L. plantarum* 2/37) could also form their own biofilms in the place of the pathogenic ones. The authors concluded that these two LAB strains could be suitable for a probiotic treatment of mastitis, and proposed them for further in vivo investigations to test their potential beneficial/barrier properties on udder health.

The biofilm matrix largely accounts for the reduced efficiency of antimicrobials against the biofilm-enclosed microorganisms by delaying their diffusion, scavenging or even inactivating them, and in parallel altering the local microenvironment of the cells, resulting in their slower growth rate and stress adaptation. This is usually composed of polysaccharides, proteins, lipids, and nucleic acids. Concerning the latter, the presence of extracellular DNA (e-DNA) has been recently been reported as a substantial component of the biofilm matrices of several microorganisms. Since the matrix plays a major role in biofilm stability, keeping it close together and hydrating the microbial cells, its degradation could consist in an effective antibiofilm strategy. This could be achieved by using enzymes targeting its main components. To this direction, Sharma and Pagedar Singh [5] tested the efficiency of DNase against mono- and mixed-species biofilms of some microorganisms relevant to the food industry (i.e., *S. aureus*, *Klebsiella* spp., *Enterococcus faecalis*, and *Salmonella* Typhimurium). First, they optimized the enzymatic treatment against biofilms formed by *Pseudomonas aeruginosa* PAO1, which was used as bacterial model due to its ability to produce copious biofilm. They applied the enzyme during biofilm formation (pre-treatment), following biofilm formation (post-treatment), and both before and after (dual treatment). Pre-treatment of DNase at a concentration of 10 μg/mL reduced biofilm formation by *P. aeruginosa* at 37 °C for 24 h by 70%, with no further efficiency to be observed upon increasing the concentration of the enzyme. Interestingly, DNase was less efficient when biofilms were older (up to 96 h), indicating that mature biofilms are more resistant than those of lower age. Post-treatment for 15 min with the same concentration of the enzyme was proven to be more efficient, resulting in a 73–77% reduction in biofilm biomass, depending on the age of the biofilm (24–96 h). The concomitant presence of Mg^2+^ ions (10 mM), used as cofactors for the enzyme, resulted in 90% reduction of *P. aeruginosa* biofilm at a half concentration (i.e., 5 μg/mL) and irrespectively of the age of biofilm. No significant differences were observed between the pre-, post-, and dual-treatments on mono-species biofilms of all the other bacteria, with their susceptibility to DNase still being organism specific. In addition, DNase was less efficient against 24 h-old mixed-species biofilms compared to mono-species ones, and its efficiency was further reduced when biofilms were grown for 48 h. The authors concluded that further optimization is required before applying DNase in cleaning regimes in food industries targeting both biofilm prevention and reduction of mixed-species sessile consortia.

*Salmonella enterica* is a major foodborne pathogen, worldwide, being frequently implicated in large outbreaks. Many studies have explored its ability to produce biofilm on either abiotic or biotic surfaces and, like with other microorganisms, this is considered as an important stress adaptation strategy [6]. Paz-Méndez et al. [7] investigated the ability of 13 strains of this pathogen, isolated from poultry houses and belonging to three different subspecies (i.e., *enterica*, *arizonae*, and *salamae*) and nine different serovars (including Typhimurium, Enteritidis, Newport, Infantis etc.) to produce biofilm on two different surfaces (i.e., stainless steel and polystyrene), incubated for 48 h in four different growth media at two temperatures (i.e., 6 °C and 22 °C). The colony morphotypes of these strains and their motilities were also investigated at both temperatures. They found that the diluted laboratory growth medium favored biofilm formation, irrespective of the surface and temperature tested compared to the other media containing food residues and used to simulate growth conditions encountered in the different food industries (i.e., dairy, meat and vegetables). Nevertheless, most of the strains were still able to produce biofilm in the presence of food residues under all the tested conditions. Almost all strains (except two) produced the red, dry, and rough (RDAR) morphotype at 22 °C, whereas a soft and completely white (SACW) morphotype was apparent at the lower temperature (i.e., 6 °C). RDAR morphotype is known to arise due to the production of cellulose and curli fimbriae, which have been both described as the main extracellular polymeric substances (EPSs) of the *Salmonella* biofilm matrix. Indeed, biofilm formation was higher at 22 °C compared to 6 °C, with the exception of tomato juice, where the biomass differences were not significant. However, the fact that most of the strains were still able to produce biofilm at 6 °C implies that other components and genetic mechanisms should play a role in the transition of cells to this sessile lifestyle. Similar to the biofilm-forming capacity, the mean motility of the strains was significantly higher at 22 °C than at 6 °C. The authors conclude that *Salmonella* bacteria may use food residues to produce biofilms on common surfaces of the food chain. Further studies combining more strains and food residues should increase our knowledge on *Salmonella* biofilm behaviour in the presence of such nutrient’s sources. These are considered important since they better mimic food industry conditions, which may well differ from those encountered in the laboratory, inducing drastic implications on biofilm/cellular physiology and resistance. 

The resistance of *Salmonella* being confined in biofilm structures to disinfectants commonly used during poultry processing is surely an alarming public health issue. The review of Cadena et al. [8] examines the modes of action of various types of disinfectants (including hexadecylpyridinium chloride, peracetic acid, sodium hypochlorite, and trisodium phosphate) against *Salmonella* in either planktonic or biofilm state, and in parallel describes the mechanisms that may confer tolerance to such disinfectants and cross-protection to antibiotics. The authors conclude that poultry processors should try to use various disinfectants presenting different modes of action to limit the ability of the bacteria to adapt and display antimicrobial resistance (AMR). The use of alternative approaches, such as enzymes, bacteriophages, and quorum sensing inhibitors, may also be valuable towards the control of biofilms and food safety assurance with lower probabilities of AMR induction. In addition, since the in situ detection of biofilms is important to be able to optimize the prevention and control methods, some commercially available devices and kits that could be used for either qualitative or quantitative, direct or indirect characterization of biofilms encountered in food processing environments are reported.

This Special Issue finishes with an interesting review presenting an update on our knowledge related to *Listeria monocytogenes* biofilms in food-related environments and their implications mainly towards biocide resistance [9]. Legislation, important ecological aspects (i.e., influence of microbial interactions on resistance in mixed-species biofilms), and some potential biocontrol strategies (i.e., use of lactic acid bacteria and/or their bacteriocins, alone or in combination with other strategies) are also reported. Undoubtedly and considering the significant risk posed by this pathogen, especially against vulnerable population groups (e.g., younger, oldest, pregnant and immunocompromised), the better understanding of the various genetic and physiological underlying mechanisms leading to its antimicrobial recalcitrance, together with the influence of pre-existing resident/transient microbiota on its sessile behavior, is significant towards our efforts to develop fast, efficient, safe, and cost-effective prevention and control treatments to improve the safety of the food supply.

The role of biofilms in the development and dissemination of microbial resistance within the food industry is surely important and multifaceted. The articles presented in this Special Issue aim to contribute to understand this problem and its magnitude, making clear the need for novel efficient intervention methods. 
